# C-851-04. Determinants of Operative Intervention in Older Adults with Burn Injury

**DOI:** 10.1093/jbcr/irag033.056

**Published:** 2026-04-06

**Authors:** Ashleigh Bull, Colette Galet, Shawn Tejiram, Lucy Wibbenmeyer, Alisa Savetamal, Deepak K Ozhathil, Dhaval Bhavsar, David M Hill, Sara Higginson, Nicole M Kopari, Lauren B Nosanov, Kathleen S Romanowski

**Affiliations:** University of Iowa Hospitals and Clinics, Iowa City, IA; University of Iowa, Iowa City, IA; MedStar Washington Hospital Center, Washington, DC; University of Iowa Health Care, Iowa City, IA; Bridgeport Hospital/Yale New Haven Health, Bridgeport, CT; Akron Children's Hospital, Akron, OH; The University of Kansas Health System, Kansas City, KS; Firefighters Regional One Burn Center, Memphis, TN; Shriners Children's - Ohio, Dayton, OH; Leon S. Peters Burn Center, Fresno, CA; Emory University School of Medicine, Atlanta, Georgia; UC Davis Burn Center & Shriners Hospital for Children, Sacramento, CA

## Abstract

**Introduction:**

Older adults represent a growing proportion of burn patients and face complex surgical decision-making. While early excision and grafting are standard, advancing age, comorbidities, and reduced physiologic reserve complicate operative planning. The factors influencing whether older patients undergo surgery remain unclear. Understanding how patient characteristics, injury patterns, and frailty status affect operative selection is essential to guide treatment and align care with patient goals.

**Methods:**

We conducted a retrospective multicenter cohort study of patients ≥60 years admitted to 12 burn centers between January 2017 and December 2019. Data included demographics, comorbidities, injury characteristics, and operative status. Frailty was assessed with the Canadian Study of Health and Aging Clinical Frailty Scale. Univariate and multivariate analyses identified factors associated with operative treatment. *p*<.05 was considered significant.

**Results:**

Operative data were available for 1528 patients; 784 underwent surgery. There were no significant differences in age, race, ethnicity, or homelessness between groups. Surgical patients had larger burns (6.1% vs. 2.5%, *p*<.0001), were less likely to have inhalation injury (7.9% vs. 10.8%, *p*<.0001), had lower albumin (3.1 vs. 3.5, *p*<.0001), and lower frailty scores (4 vs. 4, *p*<.0001). Males were more likely to undergo surgery (53.2% vs. 47.4%, *p*=.03). Patients with alcoholism (62.3% vs. 37.7%, *p*=.009) and hypertension (53.6% vs. 46.4%, *p*=.05) more often had surgery. Those with heart failure (39.3% vs. 60.7%, *p*=.008), smoking (45.0% vs. 55.1%, *p*=.005), functional dependence (28.6% vs. 55.1%, *p*=.0002), or respiratory disease (34.2% vs. 65.8%, *p*<.0001) were less likely to undergo surgery. In-hospital mortality did not differ, but surgical patients had improved long-term survival (54.9% vs. 45.1%, *p*=.003). On multivariate analysis, surgery was independently associated with burn size (OR = 1.07 [1.04–1.09]), frailty (OR = 0.76 [0.66–0.88]), inhalation injury (OR = 0.51 [0.28–0.92]), and respiratory disease (OR = 0.42 [0.29–0.92]).

**Conclusions:**

Surgical treatment was more likely in patients with larger burns, lower frailty scores, and without major comorbidities. Male sex, hypertension, and alcoholism increased surgical likelihood, while frailty, dependence, smoking, and respiratory disease reduced it. Although in-hospital mortality did not differ, surgery conferred improved long-term survival. These findings highlight the influence of physiologic reserve and comorbidity on surgical candidacy in older burn patients.

**Applicability of Research to Practice:**

Frailty and comorbidity assessment should be part of routine burn care to guide surgical decisions, anticipate nonoperative needs, and support shared decision-making.

**Funding for the Study:**

N/A.

